# Supplementary material for: Intradermal Immunization of Soluble Influenza HA Derived from a Lethal Virus Induces High Magnitude and Breadth of Antibody Responses and Provides Complete Protection In Vivo
Source: Vaccines (Basel). 2023 Mar 31;11(4):780. doi: 10.3390/vaccines11040780 (PMC10141624; doi:10.3390/vaccines11040780)
Supplement: Supplementary file 1 [file vaccines-11-00780-s001.zip › vaccines-2175087-supplementary.pdf]

vaccines

MDPI

Supplementary materials

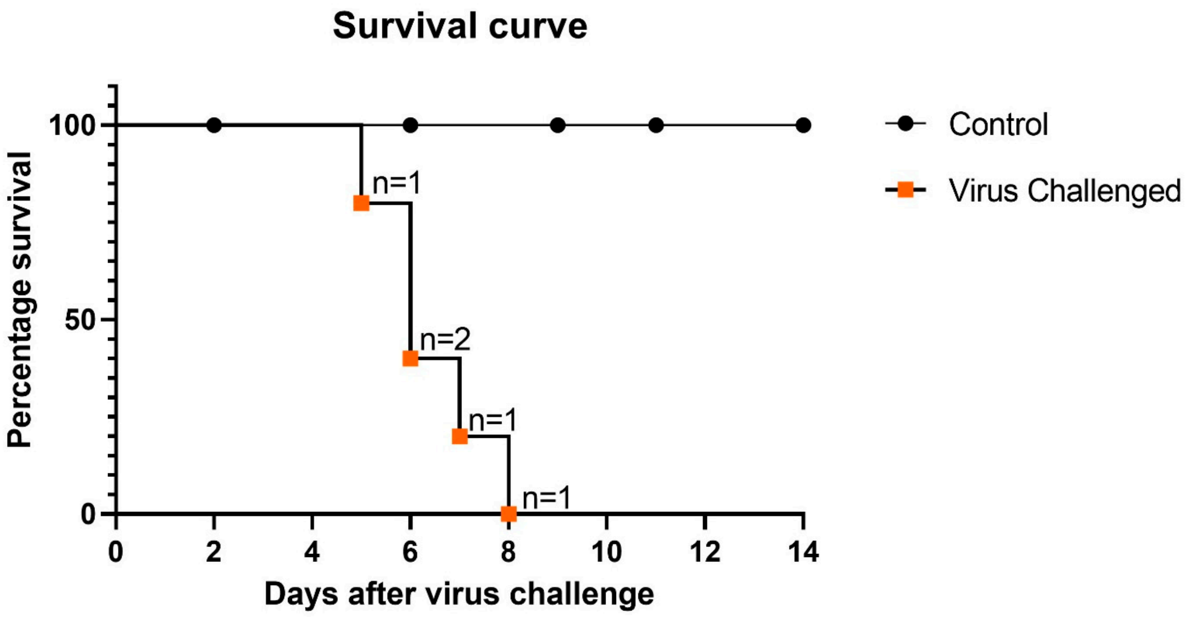

Figure S1. Survival curve showing high lethality of Inf A/Guangdong-Maonan/SWL1536/2019 (10^5 $TCID_{50}$) virus administered intranasally in 6-8 weeks old BALB/c mice (n=5) and monitored daily post challenge. All mice succumbed by day 8 post infection.

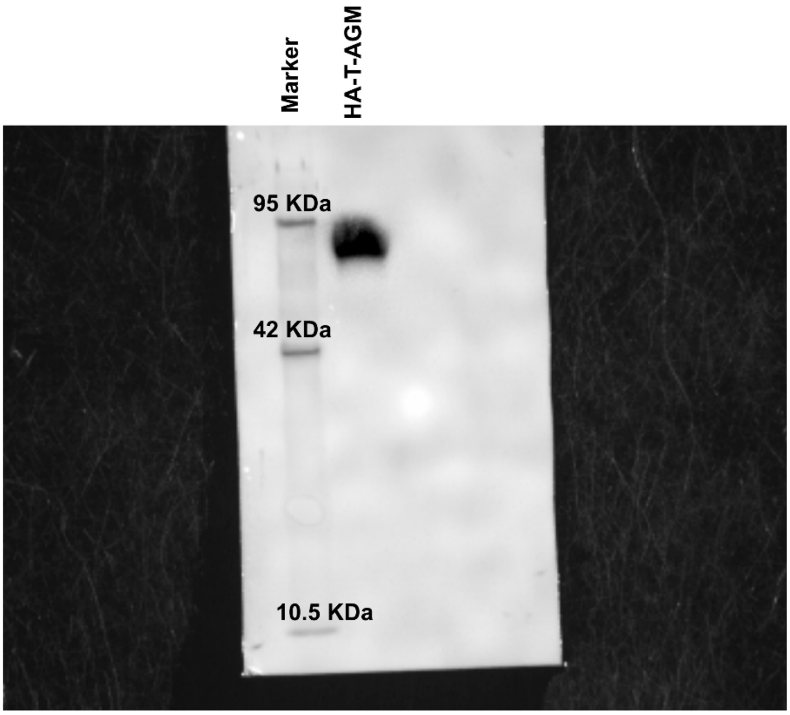

Figure S2. Original blot of HA-T-AGM protein probed with FR572 mAb as primary antibody. Intensity calculated as per software ImageJ is 50400.19 and ratio when compared to the other band on blot developed with mice sera antibody is 0.96. (Same blot was stripped and used to probe with mice sera as primary antibody).

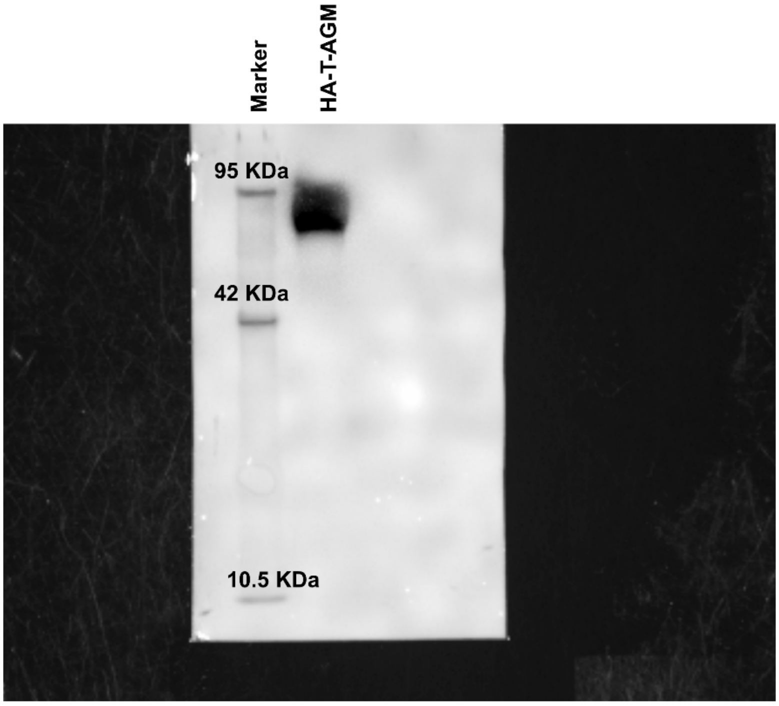

Figure S3. Original blot of HA-T-AGM protein probed with anti-HA-T-AGM mice sera as primary antibody. Intensity calculated as per software ImageJ is 48430.11 and ratio when compared to the other band on blot developed with FR572 mAb is 0.96. (Same blot was first used to probe with mAb FR572 sera as primary antibody).

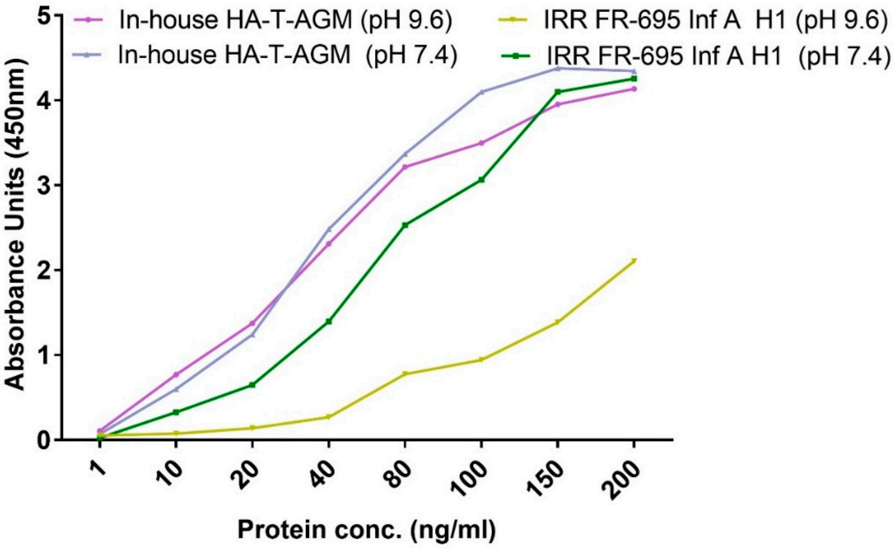

Figure S4. ELISA results showing the extent of binding of HA-T-AGM protein at difference coating concentrations reflected by the absorbance Units at 450 nm and also at two different pH buffers for antigen coating, showing a lower pH 7.4 being more favourable for readouts than buffer with pH 9.6. The binding capacity is also compared with commercially available H1 HA protein and results show slightly higher binding potential by our recombinant HA-T-AGM protein.

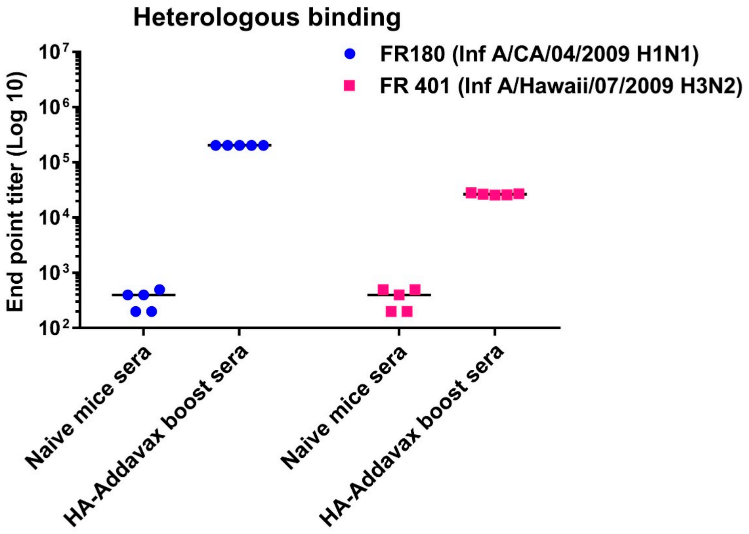

Figure S5. ELISA showing binding of anti-HA-T-AGM boost sera with heterologous virus antigens of subtypes H1N1(FR180) and H3N2(FR401) indicating cross reactivity.

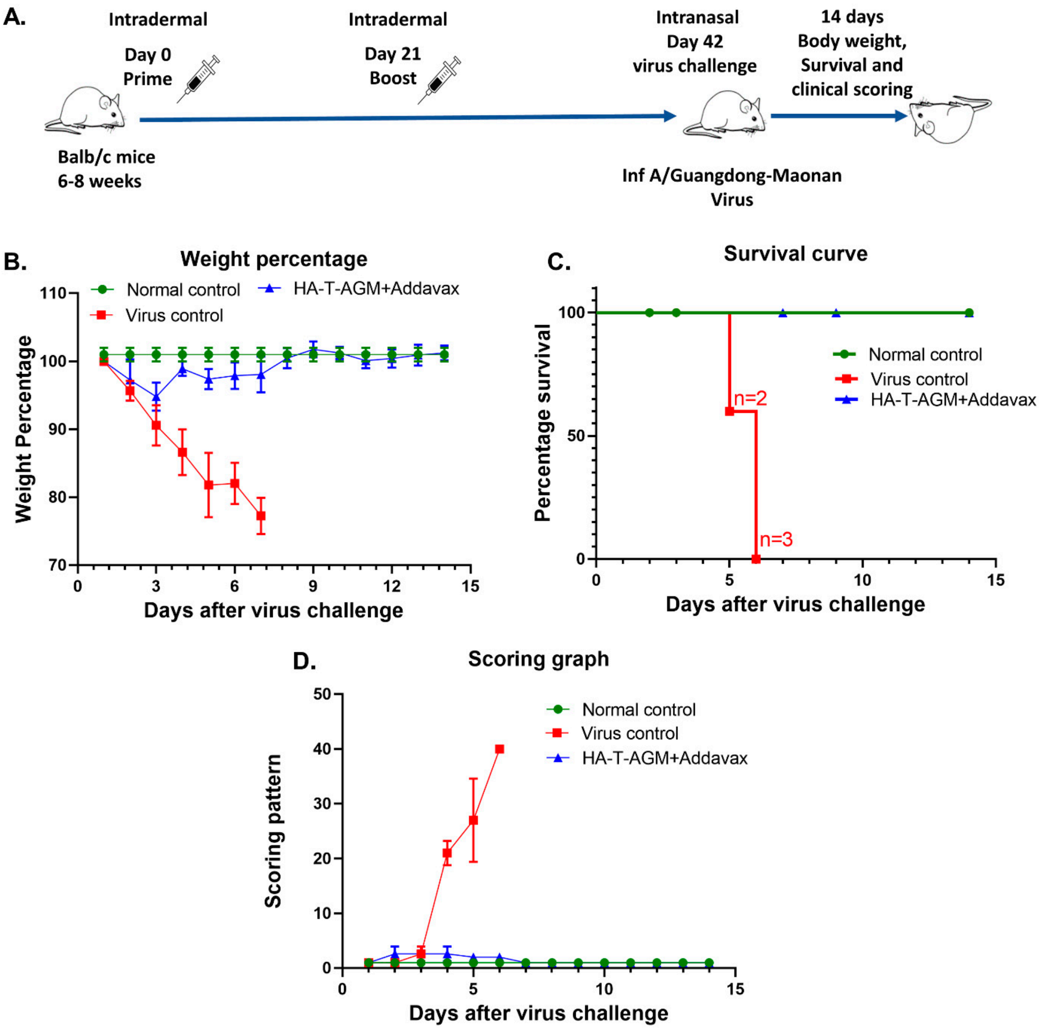

Figure S6. A: Schematic for immunization schedule with antigen HA-T-AGM **B–D**: Post virus challenge studies showing weight percentage change, survival curve and scoring graph indicating protection of the immunized group against the homologous virus challenge.

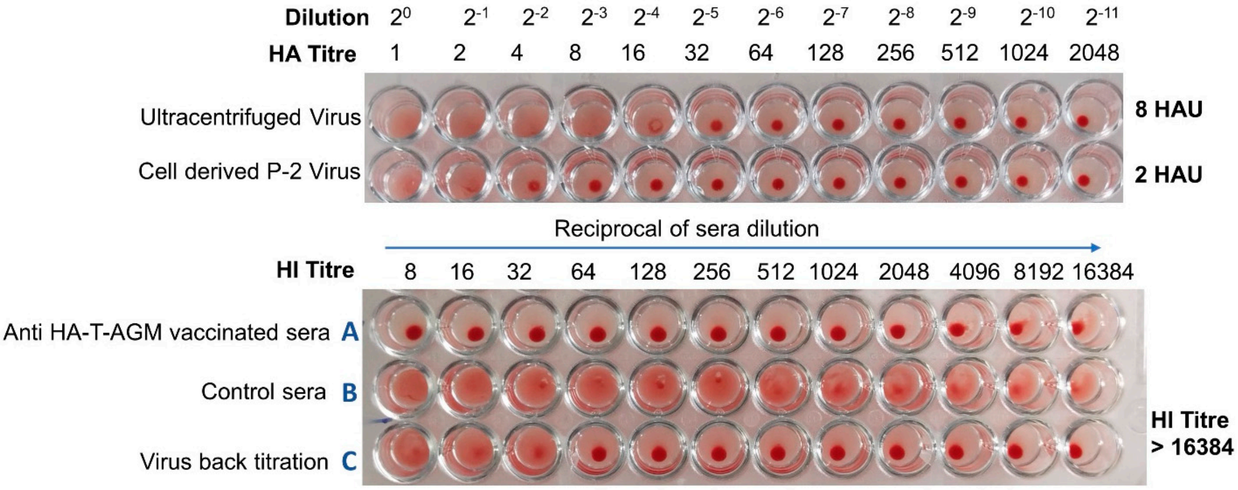

Figure S7. Plate showing Hemagglutination inhibition of homologous sera against Influenza A Guangdong Maonan virus using 4 HAU. HI titre is >16384.

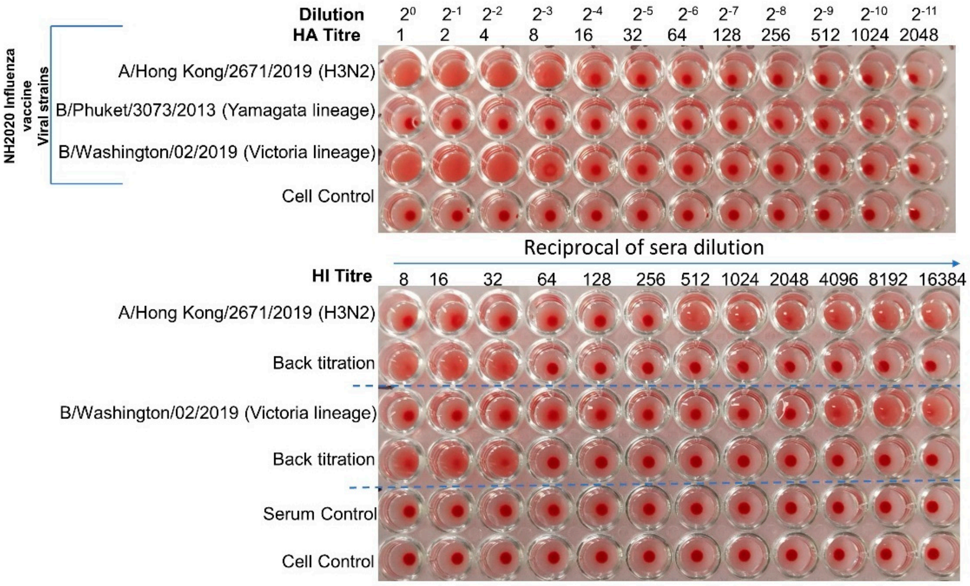

Figure S8. Hemagglutination inhibition (HI) assay by HA-T-AGM boost sera against the other NH2020 influenza vaccine component suggested virus strains showing protection against H3N2 strain as well as against type B influenza virus (Victoria lineage). HI Titer against A/Hong Kong/2671/2019(H3N2) was recorded 256 and for B/Washington/02/2019 (Victoria lineage) it was seen as 2048.

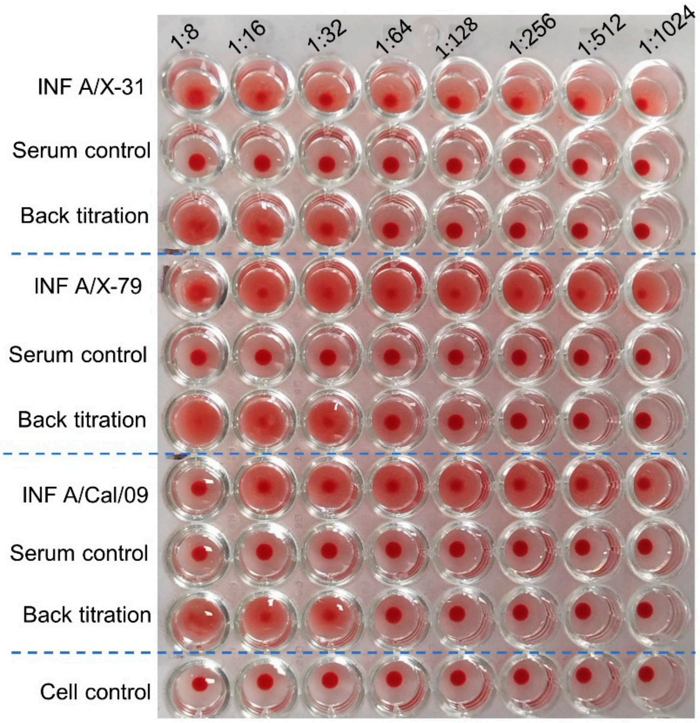

Figure S9. Plate showing HI titer of Anti-HA-T-AGM vaccinated sera against heterologous strains of virus (HI titer for Inf A/H1N1/Cal 04; Inf A/H3N2/X-31 and Inf A/H3N2/X-79 was ≤ 1024).

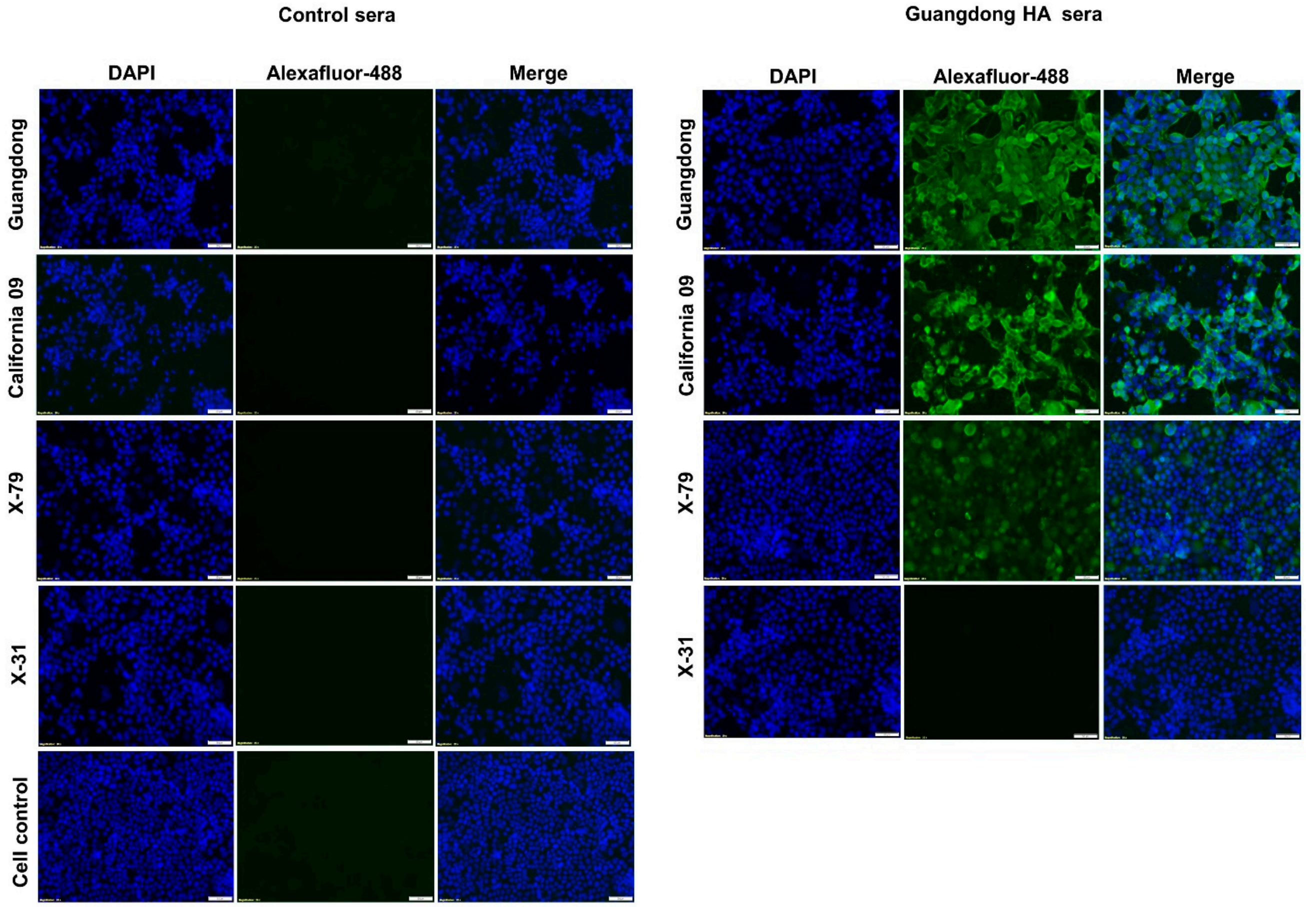

Figure S10. Influenza A/Guangdong HA- anti-sera cross reactivity with other Influenza viruses as shown with Immunofluorescence assay. MDCK London cells were seeded and infected with different influenza viruses at an MOI of 0.1. After 24 hours p.i., cells were fixed and stained with Guangdong anti-HA sera and secondly with anti-mouse Alexa fluor 488. Images were taken using Olympus fluorescent microscope at 20X magnification.

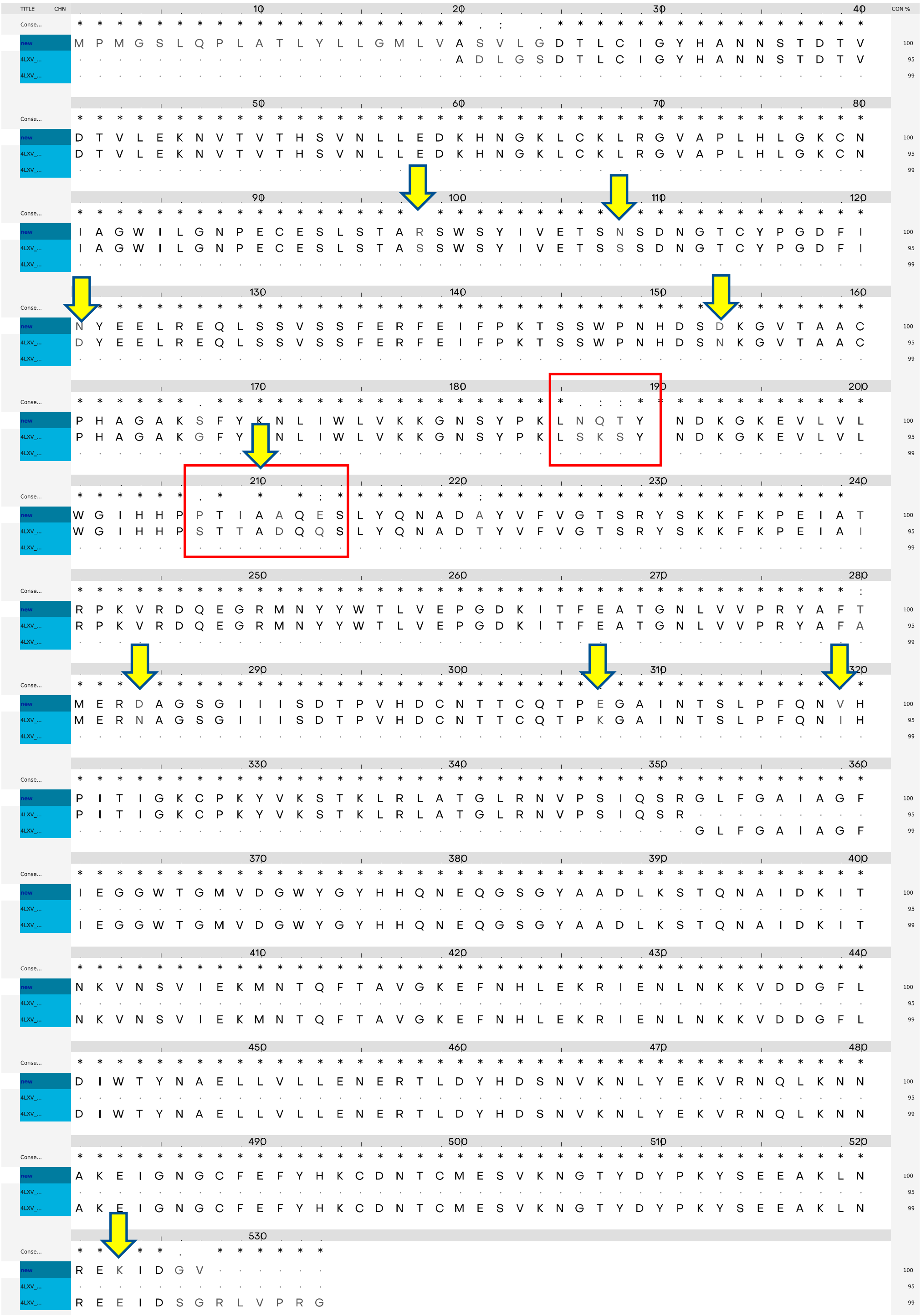

Figure S11. Sequence alignment: Sequences of target (AGM) and template (4LVX) was aligned using Schrodinger. The residual changes are highlighted by arrow (yellow) and major change is shown by box (red).
